# Inverted internal limiting membrane flap technique and an autologous platelet concentrate to treat an Nd:YAG laser-induced macular hole: A case report

**DOI:** 10.1097/MD.0000000000018185

**Published:** 2019-11-22

**Authors:** Young Hoon Yang, Young Taek Chung, Bu Ki Kim, Jun Hyung Moon, Su Joung Mun

**Affiliations:** Onnuri Eye Clinic, 325, Baekje-daero, Wansan-gu, Jeonju-si, Jeollabuk-do, Republic of Korea.

**Keywords:** autologous platelet concentrate, internal limiting membrane, inverted flap technique, macular hole, Nd:YAG laser

## Abstract

**Rationale:**

Nd:YAG laser-induced macular holes (MHs) feature more extensive anatomical defects and worse functional outcomes than idiopathic MHs. Although new treatment options for large refractory MHs have been suggested, the current literature on Nd:YAG laser-induced MHs suggests only conventional pars plana vitrectomy combined with internal limiting membrane (ILM) peeling, which is the same treatment as for idiopathic MHs.

**Patient concerns:**

A 40-year-old dermatologist was referred to us because of a sudden decrease in visual acuity following exposure to a floor-tile-reflected single-shot Nd:YAG laser beam while not wearing protective goggles.

**Diagnoses:**

An Nd:YAG laser-induced MH was diagnosed based on fundoscopy and optical coherence tomography (OCT).

**Interventions:**

Pars plana vitrectomy using an inverted ILM flap technique and autologous platelet concentrate (APC) was performed.

**Outcomes:**

Postoperative spectral domain OCT and en-face OCT showed “U-shaped” closure of the MH and a decreased ellipsoid zone defect, while the best-corrected visual acuity improved from 20/500 to 20/25.

**Lessons:**

The inverted ILM flap technique combined with APC is an effective option to achieve successful MH closure and visual improvement in patients with an Nd:YAG laser-induced MH.

## 1 Introduction

A macular hole (MH) is a recalcitrant ocular injury that can be induced by Nd:YAG laser exposure and can cause significant visual deterioration. Nd:YAG laser-induced MHs tend to occur in relatively young people, and cause more severe anatomical disruption (and therefore have a greater socioeconomic impact) than idiopathic MHs. Persistent postoperative outer retinal defects with limited visual recovery are common; a treatment that provides a high success rate and rapid recovery allowing an early return-to-work for patients is required.

To improve closure rates and functional outcomes in large refractory MH patients, several treatment options have been suggested, and there have been several recent reports of successful closure using the inverted internal limiting membrane (ILM) flap technique.^[1–4]^ In addition, autologous platelet concentrate (APC) has been used to improve the MH closure rate.^[5]^ Thus, it might be assumed that combination of these two techniques might be more effective for closure of large refractory MHs, but the application of both techniques has rarely been reported, with only Finn et al^[6]^ recently reporting a case of a large traumatic MH that was successfully closed using the combination of an ILM flap and APC. Therefore, we report the first application of both an inverted ILM flap and APC in an Nd:YAG laser-induced large MH, which resulted in successful MH closure and significant visual improvement.

## 2 Case report

This case was performed in accordance with the tenets of the *Declaration of Helsinki*. Written informed consent was obtained from the patient for publication of this case report and any accompanying figures.

A 40-year-old dermatologist was referred due to a sudden decrease in visual acuity in the right eye (OD) of 3 days duration. He reported exposure to a floor-tile-reflected single-shot Nd:YAG laser beam while not wearing protective goggles. The laser settings were: wavelength, 1064 nm; pulse energy, 7 J/cm^2^; duration, 10 ns; and spot size, 3 mm.

**Figure 1:**
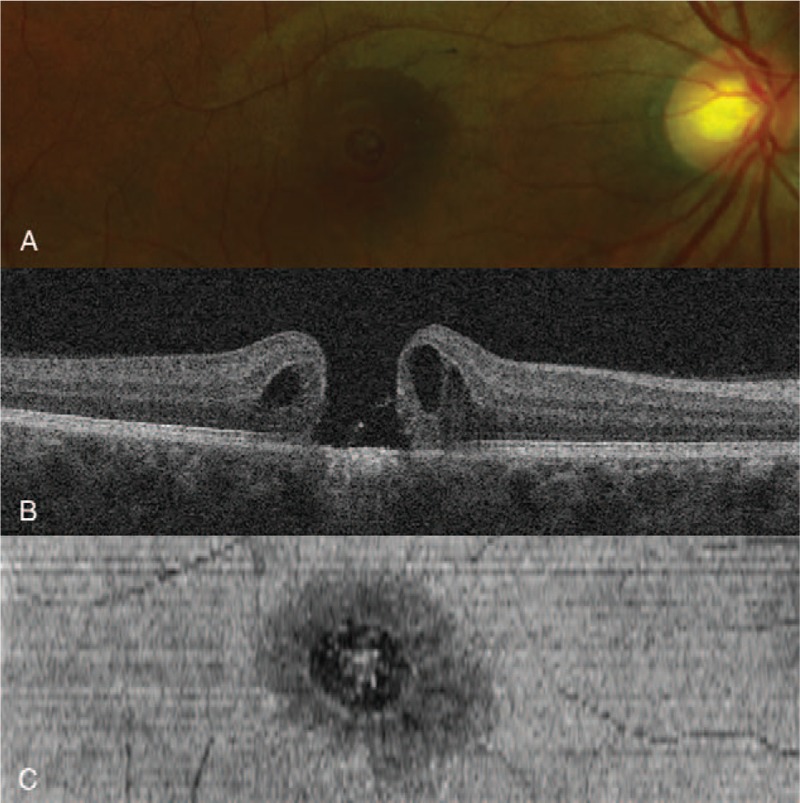
(A) Fundus photography of the right eye 2 weeks after initial laser exposure demonstrating a full-thickness macular hole (MH). (B) Spectral domain optical coherence tomography (OCT) indicated a 652-μm full-thickness MH, cystoid macular edema in the outer nuclear layers, and disruption of the adjacent external limiting membrane, ellipsoid, and photoreceptor zones. (C) En-Face OCT indicated centrifugal ellipsoid zone disruption extending over the MH.

At initial presentation, his best-corrected visual acuity (BCVA) OD was 20/100. A fundus examination and spectral domain optical coherence tomography (OCT) OD demonstrated a macular disruption filled with hemorrhagic and coagulative debris. Two weeks later, the hemorrhage had been absorbed, revealing a full-thickness MH 652 μm in size (Fig. 1). Despite strong advice to undergo surgical repair, the patient initially hesitated due to his concerns regarding postoperative visual improvement. He decided to undergo surgery only after the BCVA had dropped to 20/500 at 4 weeks after the injury.

**Figure 2:**
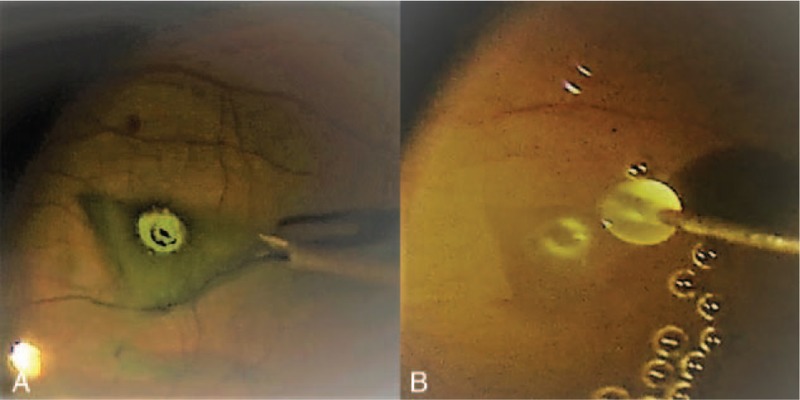
Intraoperative photographs showing the surgical procedure of the inverted internal limiting membrane (ILM) flap technique and autologous platelet concentrate (APC). (A) After the peeled ILM was trimmed with a vitreous cutter, the superior half of the peeled ILM was inverted inferiorly with intraocular forceps to cover the entire MH, working as a single-layer flap. (B) After air-fluid exchange, 0.3 ml of APC was injected to submerge the inverted flap. The mixture of the inverted ILM flap and APC formed a macular plug shortly afterward and stabilized the flap, thus preventing spontaneous detachment or unfolding.

Surgery was performed using a 25-gauge vitrectomy system. After staining with indocyanine green, the ILM was peeled centripetally approximately 4DD in diameter until it was attached only at the edge of the MH. The lower half of the peeled ILM was then removed while the remaining upper half was inverted gently, covering the MH as a single-layer flap (Fig. 2A). After air-fluid exchange, 0.3 ml of APC was injected over the posterior pole, submerging the flap (Fig. 2B). Finally, gas tamponade with 14% C3F8 was performed. The patient lay in the supine position for the first 12 hours after surgery and then remained prone over the next 7 days.

**Figure 3:**
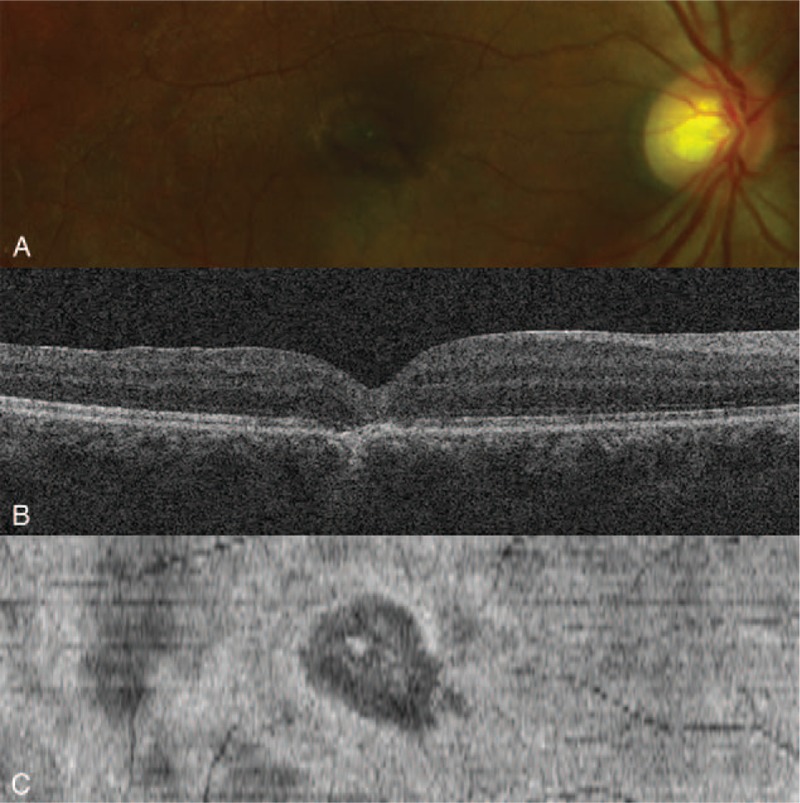
(A) Fundus photography 6 months following surgery. (B) Spectral domain OCT indicated closure of the MH with a normal foveal contour and without a persistent outer retinal defect. (C) En-Face OCT indicated decreased ellipsoid zone disruption.

At 1 month after surgery, the patient had a BCVA OD of 20/50 and spectral domain OCT showed closure of the MH. At 6 months after surgery, the BCVA had improved to 20/25. Spectral domain OCT showed a “U-shaped” closure of the MH, while en-face OCT showed a decreased ellipsoid zone defect (Fig. 3).

## 3 Discussion

An Nd:YAG laser-induced MH requires active treatment as it tends to occur in relatively young individuals and the damage is more severe than in idiopathic MHs. Nd:YAG laser-induced MHs develop as a result of laser exposure and subsequent photomechanical and photothermal damage to the macula.^[7,8]^ Laser-induced acoustic shock spreads centrifugally and causes secondary damage to the surrounding retina, retinal pigment epithelium, and choroid, resulting in extensive ellipsoid and photoreceptor zone damage beyond the size of the MH. This extensive damage may lead to persistent outer foveal defects and limited postoperative visual improvement. Therefore, treatment of Nd:YAG laser-induced MHs requires a better surgical method than the conventional ILM peeling technique used for idiopathic MHs.

In this case, use of the inverted ILM flap technique and APC led to successful MH closure and significant visual improvement, similar to the report of Finn et al.^[6]^ These good results can be attributed to the advantages of the inverted ILM flap technique, which acts as a scaffold for Müller cell activation and subsequent gliosis, combined with the advantages of APC, which enhances glial cell proliferation. As the ILM flap and APC together form a macular plug that seals the MH, the healing process is accelerated. Moreover, stabilization of the flap by the glue-like nature of the APC and appliance of the superior ILM flap may also contribute to good results. We consider that using a superior ILM flap has the advantage of reducing sagging and stabilizing the flap under the effect of gravity even when the patient is in an upright position.

In addition to a good visual prognosis, postoperative OCT scans showed U-shaped closure of the MH and a decreased ellipsoid zone defect. While the scan revealed a foveal residual neuroretina approximately 200 μm thick, no definite outer foveal defect was present. These results correspond to the report of Stein et al^[9]^ indicating that persistent postoperative damage to outer retinal structures may be a poor prognostic feature and that of Qi et al^[10]^ indicating that patients with better postoperative BCVA have a thicker foveal residual neuroretina or thinner subfoveal defect. Therefore, combined use of the inverted ILM flap technique and APC, which actively induces gliosis and restores foveal structure, may lead to a better visual prognosis in Nd:YAG laser-induced MHs.

In conclusion, successful MH closure and significant visual recovery can be achieved using the inverted ILM flap technique and APC for Nd:YAG laser-induced MHs. Together, the inverted ILM flap technique and APC may enhance restoration of foveal structure and lead to a better visual prognosis.

## Acknowledgment

The authors thank Textcheck (www.textcheck.com) for English language editing.

## Author contributions

**Conceptualization:** Su Joung Mun.

**Data curation:** Jun Hyung Moon.

**Investigation:** Bu Ki Kim.

**Supervision:** Young Taek Chung.

**Writing – original draft:** Young Hoon Yang.

**Writing – review & editing:** Young Hoon Yang, Su Joung Mun.
